# Impact of ESPGHAN no-biopsy strategy on the outcome of celiac disease treatment in children

**DOI:** 10.3389/fped.2025.1546711

**Published:** 2025-03-20

**Authors:** Iulia Enache, Mariana Jinga, Alexandra Palaga, Daniel Vasile Balaban, Felicia Galos, Dhea-Maria Macovei, Alina Popp

**Affiliations:** ^1^ Department of Pediatrics, Carol Davila University of Medicine and Pharmacy, Bucharest, Romania; ^2^ Department of Gastroenterology, Dr. Carol Davila Central Military Emergency University Hospital, Bucharest, Romania; ^3^ Department of Pediatrics, Alessandrescu-Rusescu National Institute for Mother and Child Health, Bucharest, Romania; ^4^ Department of Pediatrics, Marie-Curie Emergency Children’s Hospital, Bucharest, Romania; ^5^ Celiac Disease Research Center, Faculty of Medicine and Health Technology, Tampere University and Tampere Hospital, Tampere, Finland

**Keywords:** childhood celiac disease, serology-based diagnosis, gluten-free diet, transgressions, long-term outcome, biopsy-proven diagnosis

## Abstract

**Aim:**

The European Society for Pediatric Gastroenterology Hepatology and Nutrition (ESPGHAN) allows a no-biopsy diagnostic of celiac disease under certain conditions. We assessed the impact of the diagnostic algorithm on the patient's long-term outcome by comparing the serology-based diagnosed patients to biopsy-proven ones.

**Methods:**

We reviewed the charts of children presenting with antitransglutaminase IgA titers above ten times upper limit of normal and consecutively diagnosed with celiac disease between 2010 and 2014, a time-period overlapping with ESPGHAN diagnostic guideline change in 2012. Outcome measures for no-biopsy vs. biopsy-proven diagnosed patients were clinical and laboratory findings, compliance to gluten-free diet and to regular visits after one, two and 8–10 years of follow-up.

**Results:**

Clinical and laboratory, i.e., serum chemistry and autoantibody outcome measures on gluten-free diet clearly showed worse patient healing in the 33 serology-based diagnosed children compared to the 30 biopsy-proven ones. The attendance of the follow-up visits was also higher in the biopsy group.

**Conclusions:**

Our results indicate that dietary transgressions are common in childhood celiac disease resulting in slow healing. Therefore, there is a need of improvement of the management, with special attention regarding the ESPGHAN no-biopsy criteria diagnosed patients. Our study also indicates that novel treatments adjunctive to diet are warranted in children.

## 1 Introduction

Celiac disease (CD) is a systemic autoimmune disorder, which gradually develops as a response to gluten intake in genetically predisposed individuals. Once diagnosed, the only available treatment is a strict life-long gluten-free diet (GFD), a major change in the life of the children and their families.

As patients may seem adherent to the dietary restriction, transgressions can occur at any time, even after years of treatment. Further, inadvertent gluten ingestion is common even when following a strict GFD (1). Therefore, regular follow-up of these children is critical (2). One of the main characteristics of CD is the production of specific gluten-dependent transglutaminase 2 (TG2-IgA) and endomysial (EmA) autoantibodies (3, 4). The serum identifications of the TG2-IgA and EmA are important steps of the CD diagnostic and follow-up algorithms. Starting 2012, the European Society for Pediatric Gastroenterology Hepatology and Nutrition (ESPGHAN) recommendations has removed from the CD diagnostic strategy the requirement for a small-bowel biopsy, the historical gold standard (5). This change was based on retrospective studies showing that high autoantibody titers are indicative for a severe duodenal mucosal lesion (6, 7). Further, large multicentric prospective studies confirmed these findings showing that up to 50% of the children clinically suspected for CD would not need the intestinal biopsy to complete the diagnosis (8–10). Therefore, clinical diagnosis of CD was allowed based on serology alone, in children having serum TG2-IgA titers higher than 10 times the upper limit of normal of the laboratory kit used (TG2-IgA>10xULN) provided that EmA were also positive from a different blood sample (11). A recent meta-analysis showed that the no-biopsy criteria have high positive predictive value when compared with the small bowel biopsy reference standard (12). Quantitative measurements of TG2-IgA are further recommended during follow-up visits with the expectation of full normalization by 24 months in most of the children (11).

In the present study we sought to assess the impact of the change of CD diagnostic strategies on the patient's long-term outcome regarding clinical and laboratory features, dynamics of CD serum antibody titers and adherence to follow-up appointed visits by comparing serology-based to biopsy-proven diagnosed patients.

## 2 Methods

### 2.1 Patients and study design

The study includes retrospectively collected data from the medical charts of consecutive children who were diagnosed and followed for CD at the National Institute for Mother and Child Health, Bucharest, between 2010 and 2014. This 4-year period overlapped with the 2012 guideline change of diagnostic algorithm in pediatric CD, from duodenal biopsy-proven to allowing serology-based diagnostics. All CD diagnoses were established according to valid diagnostic criteria at the time of diagnosis (5, 13). After applying our inclusion criteria, i.e., a firm, either serology-based or biopsy-proven diagnosis at the age of <16 years and having an initial serum TG2-IgA titer >10xULN, the children were divided into two study groups, the serology-based group and the biopsy-proven group. The serology-based diagnosed group patients had also a confirmatory EmA positivity and demonstrated the mandatory CD genetic risc alleles, human leukocytes antigens DQ2 and/or DQ8 (5).

### 2.2 Data collection

Clinical data were collected according to a standardized medical chart and compared between the two groups. This included, when available, duration of symptoms before the diagnosis, dietary adherence and time-length between the diagnosis and start of GFD. The family history of CD and the presence of CD associated conditions (type 1 diabetes, autoimmune thyroid disease) were recorded. The clinical and laboratory data again were collected at baseline at CD diagnosis and follow-up visits, after both 1 year and 2 years of GFD. Additionally, data were collected from everyone at their last follow-up visit in our unit. Weight and height measurements were expressed as body mass index (BMI) calculated as weight/height^2^ (kg/m^2^) adjusted for age and gender. Symptoms were divided into gastrointestinal and extraintestinal. The gastrointestinal symptoms were further divided in diarrhea, abdominal pain, constipation, vomiting and flatulence problems. Data on asymptomatic children diagnosed by screening among risk groups were collected. Further, laboratory values of serum autoantibodies and of blood biological parameters comprising hemoglobin [Hb, lower reference values (rfv) being 11 g/dl–13 g/dl according to age and gender], mean erythrocyte volume (MCV, rfv 73–95 fl), alanine aminotransferase (ALT, upper rfv 32 U/L), iron (lower rfv 8 µmol/L), ferritin (lower rfv 20 µg/L), vitamin D (lower rfv 75 nmol/L), and alkaline phosphatase (upper rfv 500 U/L) were also gathered. For the quantitative measurements of the serum TG2-IgA the same enzyme-linked immunosorbent assay kit was used at diagnosis as well as at each follow-up visit. The cut-off for positivity was set at 20 U as recommended by the kit producer (Quanta Lite, Inova Diagnostics, CA, USA). Serum EmA was assessed by indirect immunofluorescence method using the same laboratory kit at diagnosis and each follow-up visit (Nova Lite, Inova Diagnostics). The cut-off for positivity was set at a serum dilution of 1:5. Positive samples were further diluted in 1:50, 1:100, 1:200, 1:500, 1:1,000, 1:2,000 and 1:4,000 to obtain the highest positive titer. All EmA assessments were done by the same observer.

Compliance with regular follow-up visits and adherence to GFD were evaluated at each follow-up visit at 1 and 2 years after starting GFD. The adherence to the diet was assessed with the help of a questionnaire filled in by all the children or caregivers after starting the GFD. It contained 2 simple questions: *1. How long after being diagnosed with CD did you start the GFD?; 2. How frequently do you transgress from your GFD? A. Never, B. Sometimes*.

### 2.3 Statistical analysis

Statistical analysis was done using SPSS software version 20.00 (Statistical Package for Social Sciences, IBM, US). For the statistical significance of the differences between the groups the T test was used. Statistical significance was defined by *p*-value <0.05. Results are expressed as average ± standard deviation or median (range, quartiles) as appropriate.

## 3 Results

### 3.1 Baseline characteristics of patients in the diagnostic groups

The baseline characteristics for the two study groups of children are presented in Table 1. Altogether 63 out of the 82 newly diagnosed children with CD fulfilled our inclusion criteria. Of this, 63.5% were girls with no difference between the serology-based diagnosed children (*n* = 33, median age 3.3 years, range 1.1–14.5) and the biopsy-proved (*n* = 30, median age 4.8 years, range 1.2–15.9) respectively.

**Table 1:** Baseline characteristics of the 63 children with high serum antibody titres at diagnosis (TG2-IgA>10xULN).

Characteristics of the patients	Serology-based diagnosis *n* = 33	Biopsy-proven diagnosis *n* = 30	*p*-value
Age, median, (range) yrs	3.33 (1.1 to 14.5)	4.87 (1.2 to 15.9)	0.819
Girls, %	63.6	63.3	0.980
Celiac disease in family, %	6.1	16.7	0.187
Celiac disease associated conditions, %	3	20	–
BMI Z-score median (quartiles)	−1.51 (−2.7 to −0.2)	−1.5 (−2.92 to −0,15)	0.840
Clinical presentation			
Gastrointestinal, %	72.7	63.3	0.432
Diarrhea, %	39.4	53.3	0.275
Abdominal pain, %	66.7	40.7	0.499
Constipation, %	12.1	0	0.050
Extraintestinal, %	24.2	16.7	0.466
Screen-detected, %	3.0	20.0	0.033
Laboratory results			
EmA, median titre (quartiles)	1:500 (1:100–1:1,000)	1:1,000 (1:200–1:2,000)	0.28
Hb, median (quartiles), g/dl	12.5 (10.9–13.1)	12.2 (10.6–12.9)	0.36
MCV, median (quartiles), fL	77 (73.2–80.5)	79 (73–85)	0.23
Serum iron, median (quartiles), µmol/L	11 (4–16.8)	10.5 (4–13)	0.23
Ferritin, median (quartiles), µg/L	23 (7.9–39)	28 (5–50.5)	0.80
Alkaline phosphatase, median (quartiles), U/L	169 (121.5–211)	256 (238–294)	0.005
ALT, median (quartiles), U/L	34 (22.5–65)	40.5 (28–55.8)	0.67

BMI, body mass index; TG2-IgA, type 2 transglutaminase antibodies IgA; ULN, upper limit of normal; EmA, antiendomysial antibody; Hb, hemoglobin; MCV, median corpuscular volume; ALT, alaninaminotransferase.

Data was available in >90% of the cases.

All these patients having TG2-IgA >10xULN were also positive for EmA, with similar titers in both groups (Table 1). In the biopsy-proven group, 8/30 children and their parents chose to have a biopsy even though the new criteria would have allowed them to omit it. Altogether 39.7% of the newly diagnosed patients were underweight but no significant difference in growth parameters were found between the two groups, neither in age nor gender proportion. In the biopsy-proven group there was a trend towards higher prevalence of family history and CD associated conditions. Children presented mainly with one or more gastrointestinal symptoms with abdominal pain and diarrhea being the most frequent. CD patients with constipation were all in the serology-diagnosed group. No statistically significant differences in Hb levels, MCV and iron levels were observed. However, alkaline phosphatase was significantly lower at baseline in the serology-based diagnosis group as compared to the biopsy-proven diagnosis group (Table 1).

At the diagnosis of CD, anemia was similarly present in both groups (27.3% among children with serology-based diagnosis and 26.7% among those with biopsy-proven diagnosis). The median duration of symptoms before CD diagnosis tended to be higher in the biopsy-proven group (median 9 months, range 0–84 months) than in the serology-based group (median 4 months, range 0–132 months), *p* = 0.370. This information was available in the charts for 88% of the children. The median time between the diagnosis and the initiation of GFD was slightly higher in the serology-based group (2.3 months) than in the biopsy-proven group (1.6 months), *p* = 0.568.

### 3.2 One-year follow-up on GFD

**Figure 1:**
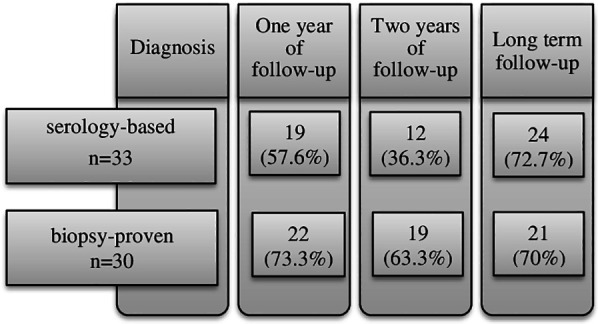
Dynamics of follow-up visits of children enrolled in the study.

Only 57.6% of children from the serology-based group responded to the GFD follow-up visit at one year, as compared to the biopsy-proven group (73.3%) (Figure 1). Most of the patients who regularly attended the follow-up visits were girls, brought up in an urban environment. Median BMI Z-score improved for both groups, in the serology group from baseline median −1.51 to −0.8 (*p* = 0.071) and in the biopsy group the result was better, from median −1.5 to −0.2 (*p* = 0.018) (Table 2). During the first year after the diagnosis, most of the patients from both groups reported improved gastrointestinal symptoms as compared to baseline, being still present in one third of the patients (Table 2). No overt clinical malabsorption syndrome was present anymore and diarrheal symptoms were rare.

**Table 2:** Follow-up characteristics in 41 children after 1 year of gluten-free diet.

Characteristics of the patients	Serology-based diagnosis *n* = 19	Biopsy-proven diagnosis *n* = 22	*p*-value
Girls, %	78.9	63.6	0.294
Urban, %	68.4	59.9	0.548
BMI Z-score median (quartiles)	−0.8 (−1.9 to 0.1)	−0.2 (−0.97 to 0.9)	0.056
Clinical presentation^a^			
Gastrointestinal, %	36.8	25	0.467
Diarrhea, %	5.3	6.3	0.944
Abdominal pain, %	26.3	18.8	0.818
Constipation, %	10.5	0	0.904
Laboratory data^b^			
TG2-IgA, mean titre (range), U	66.2 (3–200)	65 (7–173)	0.948
EmA, median titre (quartiles)	1:50 (1:5–1:1,000)	1:50 (1:5–1:100)	0.123
Hb, median (quartiles), g/dl	12.0 (11.3–13.2)	12.7 (11.9–13.2)	0.177
MCV, median (quartiles), fL	78 (72–83)	81.5 (75.5–83.25)	0.334
Serum iron, median (quartiles), µmol/L	11 (4.1–17)	8 (5.5–15)	0.874
Ferritin, median (quartiles), µg/L	27.5 (20–33.5)	59 (23–110)	0.040
Alkaline phosphatase, median (quartiles), U/L	252.5 (190–327.5)	295.5 (245.25–344.25)	0.078
ALT, median (quartiles), U/L	31 (20.5–33.5)	37 (29.5–40)	0.034

BMI, body mass index; TG2-IgA, type 2 transglutaminase antibodies IgA; EmA, antiendomysial antibody; Hb, hemoglobin; MCV, median corpuscular volume; ALT, alaninaminotransferase.

^a^ Clinical presentation data was available in all children in serology-based group and in 16 children in biopsy-proven group.

^b^ Laboratory data was available in >90% of children.

Anemia had been assessed in 16/19 children from the serology group and 14/22 children from the biopsy-proven diagnosis. No significant improvement of hemoglobin from baseline was observed in the serology group at one year of GFD, compared to the baseline level (*p* = 0.661). On the contrary, hemoglobin was higher in the biopsy-proven group compared to that from baseline after one year of GFD (*p* = 0.055) There was no statistically significant difference in the hemoglobin levels between the groups. Significantly lower ferritin in the serology group can be noted (Table 2). Alkaline phosphatase and liver enzyme ALT showed higher normal values in the biopsy-proven group after 1 year of GFD.

**Figure 2:**
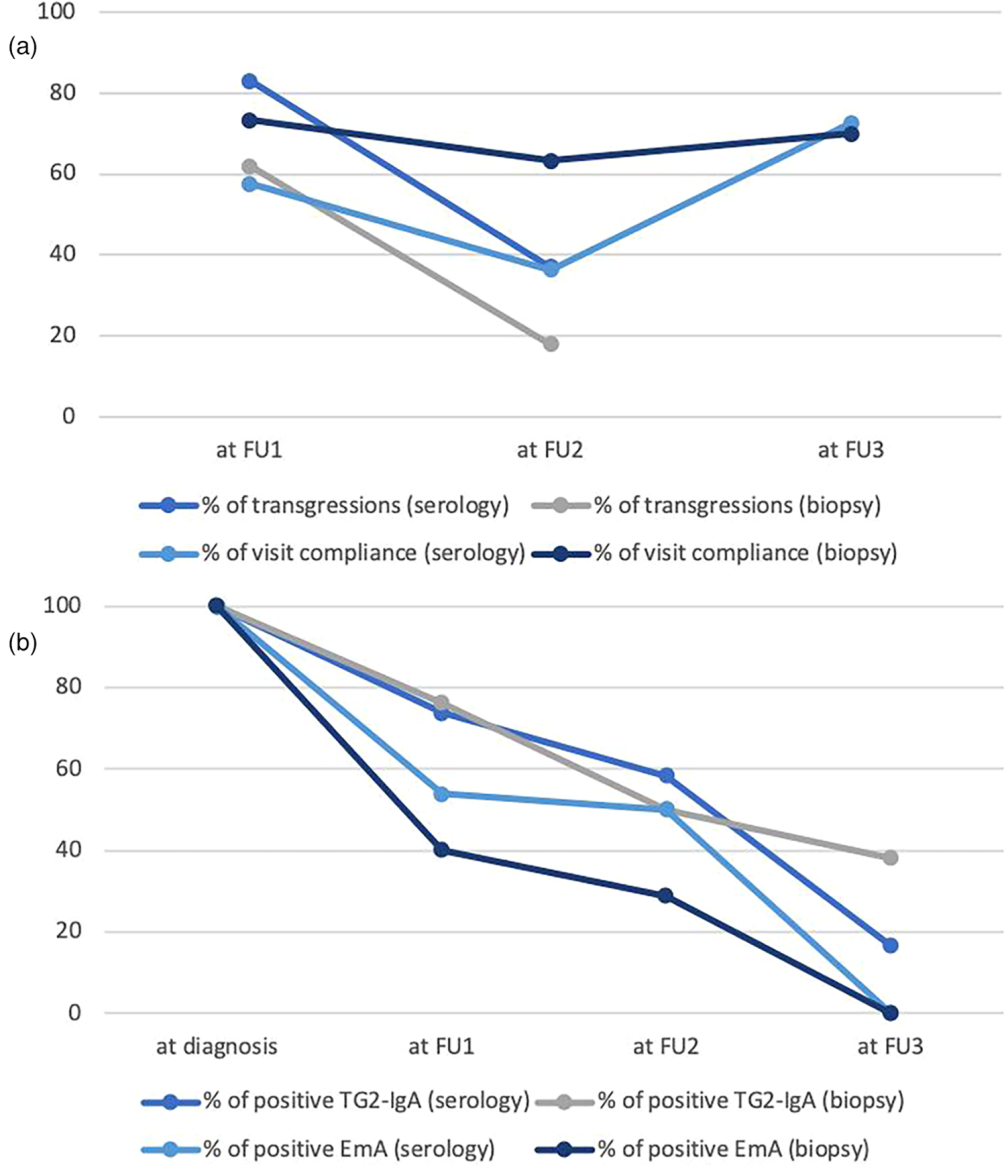
**(a)** Percentage of patients who attended the first (FU1), second (FU2) and third (FU3) follow-up visits and percentage of transgressions at FU1 and FU2 in the serology-based and biopsy-proven groups. **(b)** Percentage of positive TG2-IgA (>20 U) and EmA (<1:5) in serology-based and biopsy-proven groups at diagnosis and after the first (FU1), second (FU2) and third (FU3) follow-up.

The TG2-IgA titers had decreased from the baseline values and were similar in both groups (Table 2). However, after one year of GFD 73.7% and 76.2% of patients in the respective groups were still positive for TG2-IgA. As shown in Table 2 the EmA titers had clearly decreased as compared to baseline, but positivity was still seen in 53.9% of patients in the serology-diagnosed group and 40% in the biopsy-diagnosed group (Figure 2b).

Both groups reported occasional transgressions to GFD, 83% in the serology-based and 62% in the biopsy-proven group (Figure 2a).

### 3.3 Two-year follow-up on GFD

After 2 years of GFD, 12 children (36.3%) from the serology-based group and 19 children (63.3%) from the biopsy-proven group responded positively to the follow-up visit appointment (Figure 1). Similarly to one-year visit, most of the children compliant to the follow-up were females living in urban conditions. The BMI showed deterioration in the serology-based group (median Z-score −1.35) as compared to the initial healing at one year (−0.8) (Tables 2, 3). Median BMI was again significantly higher in the biopsy-proven group (Z-score −0.2, *p* = 0.023) with only 5.3% of these children still being underweight (BMI below percentile 5 for age and gender) as opposed to 27.3% from the serology-based group. The serology-based diagnosis group experienced more frequently gastrointestinal symptoms compared to the biopsy group (58.3% vs. 26.7%). These percentages also include overall gastrointestinal complaints, vomiting and flatulence problems, data not shown in Table 3. No overt malabsorption syndrome or diarrhea was present any more in either group. Anemia was assessed in all 12 children in the serology group and in 12/19 from the biopsy-proven group. In both groups 16.6% still had anemia. In the serology group the hemoglobin levels did not improve significantly from baseline (*p* = 0.791), while for the biopsy-proven group a significant improvement was documented (*p* = 0.025). No significant difference in ferritin levels was noted between the groups and liver enzymes were normal in both groups.

**Table 3:** Follow-up characteristics in 31 children after 2 years of gluten-free diet.

Characteristics of the patients	Serology-based diagnosis *n* = 12	Biopsy-proven diagnosis *n* = 19	*p*-value
Girls, %	75	63.16	0.508
Urban, %	75	78.95	0.806
BMI Z- score median (quartiles)	−1.35 (−3.27 to −0.22)	−0.2 (−2.13 to 0.9)	0.023
Clinical presentation^a^			
Gastrointestinal, %	58.3	26.7	0.103
Diarrhea, %	0	0	–
Abdominal pain, %	8.3	6.6	0.745
Constipation, %	16.7	0	0.069
Laboratory data^b^			
TG2-IgA, mean titre (range), U	39.7 (2–120)	31.1 (6–100)	0.504
EmA, median (quartiles)	1:5 (1:<5–1:50)	1:5 (1:<5–1:50)	0.203
Hb, median (quartiles), g/dl	12.2 (11.6–13.2)	12.9 (12.5–13.1)	0.120
MCV, median (quartiles), fL	79.5 (74.5–81.75)	80 (77–82)	0.389
Serum iron, median (quartiles), µmol/L	12 (6–18)	12 (10.5–16)	0.496
Ferritin, median (quartiles), µg/L	43 (13–58)	47 (19–60.5)	0.653
Alkaline phosphatase, median (quartiles), U/L	207.5 (175.5–349.75)	262 (248–277)	0.920
ALT, median (quartiles), U/L	25 (18–33)	31.5 (25.7–35.25)	0.125

BMI, body mass index; TG2-IgA, type 2 transglutaminase antibodies IgA; EmA, antiendomysial antibody; Hb, hemoglobin; MCV, median corpuscular volume; ALT, alaninaminotransferase.

^a^ Clinical presentation data was available in all children in serology-based group and in 15 children in biopsy-proven group.

^b^ Laboratory data was available in >90% of children.

Serum TG2-IgA and EmA titers had decreased during the GFD and there was no difference between the two diagnostic groups (Table 3). Remarkably, after two years on GFD both TG2-IgA and EmA were often still above the cut-off for positivity in both diagnostic groups; positive TG2-IgA in 58.3% patients and positive EmA in 50% patients included in the serology group; 50% and 28.6% respectively, in the biopsy group (Figure 2b). Occasional transgressions were again reported in both groups, with less frequency compared to the previous check-up visit (37% in the serology-group and 18% in the biopsy-group) (Figure 2a).

### 3.4 Follow-up after long-term GFD

Altogether 24 children (72.7%) from the serology-based diagnosis group and 21 (70%) from the biopsy-proven diagnosis group responded to the follow-up appointment at median time of 8.7 and 10 years, respectively, after diagnosis (Figure 1). Various symptoms were still reported in both groups (Table 4). In the biopsy-group no diarrhea was reported at two years on follow-up but at long-term diarrhea was again a complaint in two patients. No children had anemia anymore in either group. On long term there were no differences in biological parameters between the two groups. Serum TG2-IgA and EmA antibody titers had clearly decreased but 4 children from the serology group and 8 children from the biopsy group, altogether 27%, were still positive for TG2-IgA (Figure 2b).

**Table 4:** Last follow-up characteristics in 45 children at median time of 8.7 (serology-based group) and 10 years (biopsy-based group) on gluten-free diet.

Characteristics of the patients	Serology-based diagnosis *n* = 24	Biopsy-proven diagnosis *n* = 21	*p*-value
Girls, %	79.2	61.9	–
Age, median (quartiles), years	12.8 (10.4–15.6)	15.4 (12.95–17.8)	0.015
Gluten-free diet, median (quartiles), years	8.7 (6.33–9.6)	10 (7.5–12)	0.011
Clinical presentation^a^			
Gastrointestinal, %	19.1	13.6	0.831
Diarrhea, %	4.7	9.5	0.355
Abdominal pain, %	14.3	5.3	0.374
Constipation, %	4.7	0	0.355
Laboratory data^b^			
TG2-IgA, mean titre (range), UEmA	23.8 (4–200)1:<5	20.6 (4–115)1:<5	0.76
Hb, median (quartiles), g/dl	13.1 (12.5–13.8)	13.5 (12.65–14.7)	0.14
MCV, median (quartiles), fL	82.7 (79.5–84.3)	84.8 (80.65–88.45)	0.08
Serum iron, median (quartiles), µmol/L	13 (11–19)	17.5 (10.25–22)	0.37
Ferritin, median (quartiles), µg/L	27 (19–60)	43 (18–70)	0.26
Alkaline phosphatase, median (quartiles), U/L	211 (83–251)	100.5 (62.25–269.75)	0.15
ALT, median (quartiles), U/L	21.5 (16–24)	20 (19–28)	0.46

TG2-IgA, type 2 transglutaminase antibodies IgA; EmA, antiendomysial antibody; Hb, hemoglobin; MCV, median corpuscular volume; ALT, alaninaminotransferase.

^a^ Clinical presentation data was available in 21 children in serology-based group and in 19 children in biopsy-proven group.

^b^ Laboratory data was available in >90% of children.

The children from the biopsy-proven diagnosis group were compliant with the follow-up program significantly longer than the children diagnosed based solely on serology. In the biopsy group 15/30 (50%) children did not miss any follow-up appointment, having yearly evaluations up to the age of 17, when the transfer to the adult health care system usually begins. The remaining six children responded discontinuously to the follow-up appointments up to their respective last follow-up. The overall age at the latest follow-up evaluation was 15.4 years old in the biopsy group. Ten children/33 (30%) diagnosed based on the serology positively responded to all yearly follow-up appointments, the rest of them were discontinuously evaluated. The median age of the total group at their latest follow-up was 12.8 years old.

## 4 Discussion

Our results show that the change of diagnostic strategy from mandatory biopsy-proven to serology-based alone in 2012 (5) did in fact have an impact on patient behavior and different outcome measures both at short and long-term GFD in our present routine clinical diagnostic and follow-up study.

We noticed an overall difference in the responses to the follow-up appointments in our children and families as seen in Figure 1, the children diagnosed by biopsy were more active and responded positively to the follow-up visit appointments after one and two years on GFD. This can be explained by the burdensome longer duration of symptoms between clinical onset and diagnosis in the biopsy-proven patients, also by the greater impact of the more complex approach associated with upper gastrointestinal endoscopy at diagnosis. Further, 8 parents with serology-based diagnosis in their child demanded an endoscopy with biopsies for the diagnosis. Proof of gluten-induced small bowel injury might be a stronger driver for perceiving CD as a lifelong disease which warrants continuous follow-up, compared to serology-based only. It is of note that children diagnosed by biopsy started the diet sooner after the completion of the diagnosis than children diagnosed based on serology alone.

Our results also suggest that screen-detected children, having family members with CD, are more likely to opt for biopsy at diagnosis. We clearly need to put more effort into how to deliver the message regarding different diagnostic strategies used in CD and into the importance of follow-up in general but especially into our instructions on follow-up to serology-based diagnosed children. When looking at symptoms and signs of CD, gastrointestinal symptoms tended to be more frequent in the serology-based diagnostic group compared to the biopsy-proven one during the GFD. Furthermore, the nutritional status in general was inferior in this group as compared to the biopsy-proven group. In fact, the BMI of the serology group patients had even deteriorated at the two-year follow-up visit, and Hb values showed no improvement. We cannot identify the cause of the differences between the diagnostic groups, we can only assume that those in the biopsy group paid more attention to the children's daily nutrition, also that they potentially had less transgressions in their GFD.

When evaluating again the results of gluten-dependent celiac-type autoantibodies, TG2-IgA and EmA-IgA, and finding the high percentages of positivity on follow-up, it seems clear that the diet was all but gluten-free after one and two years of treatment. We can conclude that we did not fulfill the ESPGHAN expectation of full normalization of antibodies by 24 months in most of the children (11). However, it is known that the time required for negative seroconversion is longer for children with high titers at diagnosis and that normalization can take even longer than the expected time-length of 18–24 months of dietary elimination of gluten (14–16). The strength of our study is again the use of the same reagent kits through the time-points of diagnosis and follow-up visits.

Nevertheless, the proportion of children with persistent positive titers (51.6% for TG2-IgA and 37% for EmA) after 2 years of GFD is clearly higher than that reported in studies from other centers (14, 16–18). As mentioned, undetected transgressions but also suboptimal adherence to the diet as well as the lower accessibility to the specific gluten-free products may all together be behind the present results (1, 19). In fact, we still observed positivity for TG2-IgA in one third of the patients tested after following 8–10 years of GFD. It is of note that poor adherence to the GFD was reported with higher frequency in the group diagnosed solely by serology. This result supports earlier published assessment on the low accuracy of self-declared (by the children of their families) level of adherence and the need of dietary assessment by a health professional (20, 21). The limitation of our present study is that we lack detailed dietary assessment, this has not been done in our real-life routine clinical follow-up. Further studies are warranted using validated questionnaires to address dietary adherence. We note that half of the children declaring occasional transgressions had normal TG2-IgA titers. A promising biomarker for objective assessment of GFD adherence is the measurement of stool and urine excretion of gluten immunogenic peptides indicating continuous ingestion of gluten-containing food (22). This agrees with the meta-analysis results of Silvester et al. in 2017 (23) where it was shown that occasional transgressions are not detected by serological assessments.

The review article by Besser and Khosla in 2023 (24) presents the current understanding of CD pathogenesis and how this knowledge is being harnessed for therapeutic design and development. In adults, proof-of-concept studies for the use of several novel investigational medical products, future drug candidates using the gluten challenge design, have been presented. Today, all companies striving to develop novel treatments are trying to develop the drug for the real-life situation targeting patients already on GFD, those who still experience gluten-triggered symptoms and/or with duodenal mucosal inflammation and morphological injury. Towards this background, we feel our clinical study gives strong indication for the need of novel treatments in children suffering from CD, first as an adjunctive therapy to GFD. Nonetheless, further research needs to focus on the selection of the children which would benefit from a potential novel treatment and how this would integrate into standard care of dietary treatment. However, it should be noticed that we did not perform re-endoscopies and duodenal biopsies on our patients, but our clinical follow-up study results indicate mucosal injury to be present in our children. In a similar study, Leonard et al. 2017 (25) showed persistent enteropathy despite maintaining a GFD to be present in 20% of the children. They noticed again that neither the presence of symptoms nor positive serology were predictive of a patient's histology at the time of repeat biopsy. The authors used a qualitative Marsh grouping instrument to assess mucosal injury, known to be heavily observer dependent, as was the case in our large ESPGHAN multicenter study (9). When we again used standard operating procedures for morphometry, i.e., quantitative measurements of duodenal morphology and inflammation, baseline histology in therapeutic trials in adults revealed villus atrophy in most patients with CD who appear well controlled on GFD, i.e., symptom free and negative for TG2-IgA (26, 27). Also in children quantitative biopsy reading is essential (28). The present study findings clearly indicate that children on long-term GFD should be revisited as to their duodenal mucosal status by using quantitative morphometric tools. On long term gluten-induced small intestinal mucosal inflammation and morphological injury may cause complications to the celiac sufferers. Real-life GFD does not seem to be gluten-free in our country but neither in the USA, potentially globally (1, 25). Adjunctive therapies to GFD are warranted also in children.

A limitation of our study is the retrospective nature of it, resulting in many children and their families not complying with the invitations to the follow-up visit. On the other hand, we used this as an outcome measure in routine clinics as a strength and noticed that especially serology-based diagnosed patients need extra support and explanations as to their need of GFD and follow-up. Similarly, other studies have reported the lack of follow-up to be 14%–57% (15, 29–31), but we feel the no-biopsy diagnosed patients need our focus at present. No-biopsy strategy has been today adopted by the health-care, both in children and adults (32). Our findings indicate that in clinical practice of celiac disease management in children improvement should be focused on the follow-up of the patients. Potential interventions in order to improve compliance in these patients include regular nutritional and also possibly psychological assessments to address the potential contributing factors to their loss of adherence to the GFD and follow-up. Earlier and more frequent follow-up controls during the first two years on GFD might also improve compliance. We tried to minimize potential biases by exclusively selecting patients diagnosed according to the current at date ESPGHAN criteria, including demonstrating the presence of the CD genetic risk alleles in the serology-based group. Another limitation of the present study is represented by the small number of participants, studies on larger samples are therefore needed in order to further compare the impact of the two diagnostic strategies on the outcome of childhood CD.

In conclusion, our results indicate that serology-based diagnosis leads to slower mucosal healing, higher dietary transgressions, and lower follow-up compliance compared to biopsy-proven cases. There is a need for improvement of the management, with special attention regarding ESPGHAN no-biopsy diagnosed patients. Further long-term outcome studies are warranted to establish the best follow-up system for childhood CD. Overall, also in children, novel treatments adjunctive to GFD are needed.

## Data Availability

The data analyzed in this study is subject to the following licenses/restrictions: Data is not publicly available due to privacy and ethical reasons. Requests to access these datasets should be directed to Alina Popp, alina.popp@tuni.fi.
